# Are Chinese pediatricians missing the opportunity to help parents quit smoking?

**DOI:** 10.1186/s12887-016-0672-0

**Published:** 2016-08-20

**Authors:** Jing Liao, Jonathan P. Winickoff, Guangmin Nong, Kaiyong Huang, Li Yang, Zhiyong Zhang, Abu S. Abdullah

**Affiliations:** 1 Department of Pediatrics, The First Affiliated Hospital of Guangxi Medical University, Nanning, Guangxi 530021 China; 2 MGH Center for Child and Adolescent Health Research and Policy, Harvard Medical School, Boston, USA; 3 School of Public Health, Guangxi Medical University, Nanning, Guangxi 530021 China; 4 Duke Global Health Institute, Duke University, Durham, NC 27710 USA; 5 Global Health Program, Duke Kunshan University, Kunshan, Jiangsu Province 215347 China; 6 Department of Medicine, Boston Medical Center, Boston University School of Medicine, Boston, Massachusetts USA

**Keywords:** Pediatrician, 5A’s, Smoking cessation, Counseling, Chinese

## Abstract

**Background:**

Secondhand smoke (SHS) exposure of children due to parental tobacco use is a particularly prevalent health issue and is associated with adverse health outcomes. Following the US Clinical Practice guidelines, pediatricians in the United States deliver 5A’s (ask, advise, assess, assist, and arrange) counseling to smoking parents which has proven to be effective. We examined Chinese pediatricians’ adherence to the clinical practice guidelines for smoking cessation (i.e. 5A’s counseling practices) with smoking parents, and identified factors associated with these practices.

**Methods:**

A cross-sectional paper-and-pencil survey of pediatricians was conducted in twelve conveniently selected southern Chinese hospitals. Factors associated with any of the 5A’s smoking cessation counseling practices were identified by logistic regression.

**Results:**

Of respondents (504/550), only 26 % routinely provided 5A’s smoking cessation counseling to smoking parents. More than 80 % of pediatricians didn’t receive formal training in smoking cessation and had not read China smoking cessation guidelines; 24 % reported being “very confident” in discussing smoking or SHS reduction with parents. Pediatricians who had never smoked (OR: 2.29, CI:1.02-5.12), received training in smoking cessation (OR: 2.50, CI:1.40-4.48), had read China smoking cessation guidelines (OR: 2.17, CI:1.10-4.26), and felt very (OR: 7.12, CI:2.45-20.70) or somewhat (OR: 3.05, CI:1.11-8.37) confident in delivering cessation counseling were more likely to practice 5A’s. Pediatricians who reported “it is hard to find a time to talk with parents” (OR: 0.32, CI: 0.11-0.92) or “lack of a standard of care requiring pediatricians to provide smoking cessation or SHS exposure reduction intervention” (OR: 0.45, CI: 0.21-0.98) as a barrier were less likely to follow the 5A’s guidelines.

**Conclusions:**

Smoking cessation counseling to address parental smoking is infrequent among Chinese pediatricians. There is a need to develop and test intervention strategies to improve the delivery of 5A’s smoking cessation counseling to parental smokers.

## Background

Assisting smoking parents to quit and eliminating secondhand smoke (SHS) exposure of children is a global health priority. SHS exposure of children due to parental tobacco use is a particularly prevalent health issue in China [1] and is associated with adverse health outcomes [2–5]. The high prevalence of adult smoking in China (52.9 % in male and 2.4 % in female) reflect the fact that many children are exposed to SHS from parental smoking at home, underscoring the need for urgent public health intervention [6]. However, identifying appropriate opportunities to assist smoking parents in quitting has been a challenge. Parental smokers often see their child’s doctor more frequently than their own, with an average of over 10 visits in the first two years of a child’s life [7, 8]. Due to China’s one child policy, most Chinese parents have only one child, leading to enhanced attention to health-related concerns and frequent pediatrician visits. A child’s clinic visit could be a teachable moment to address SHS exposure of children and parental smoking cessation [9]. In earlier studies, the majority of smoking parents believed that talking about parental smoking cessation [10, 11] or SHS exposure reduction to child [12] was the responsibility of pediatricians. Pediatricians are in a key position to influence parental smoking behavior in a repeated and consistent manner [13]. In the United States, the American Academy of Pediatrics recommends that pediatricians follow clinical practice guidelines for smoking cessation and provide SHS exposure reduction and smoking cessation counseling to parents [14–17]. The clinical practice guidelines recommended five specific steps for brief counseling interventions by providers, commonly referred to as the 5A’s (ask, advise, assess, assist, and arrange) [17]. Healthcare providers are encouraged to help smokers quit smoking by implementing the 5A’s strategies as follows: *Ask* - Systematically identify all tobacco smokers at every visit; *Advise* – Strongly urge all tobacco users to quit; *Assess*- Determine the patient’s willingness to make a quit attempt; *Assist* – Aid the patient in quitting by offering counseling and/or pharmacotherapy; *Arrange* – Arrange for follow-up. These five steps are designed to be brief, but the full implementation of all 5A’s provides the necessary tools for parents to quit successfully [18].

Health systems in many high income countries have implemented the 5A’s and reported its effectiveness [19–23]. Little is known about the use of 5A’s by healthcare professionals in low- and middle-income countries. Following the 2006 US clinical practice guidelines [14], China developed Chinese smoking cessation guidelines in 2007 [24] and explored different initiatives to promote their use by healthcare professionals. However, adherence to the 5A’s by different types of healthcare professionals is not known. No previous studies in China have examined the delivery of 5A’s within the pediatric setting. Given that pediatricians are in a key position to address parental smoking cessation by incorporating the child’s health concerns as motivation [5], understanding the current pattern of 5A’s use by pediatricians and identifying the associated facilitators and barriers may help develop targeted initiatives to promote parental smoking cessation in this clinical context. We assessed the 5A’s counseling practices of Chinese pediatricians and examined the factors associated with 5A’s smoking cessation counseling practices.

## Methods

### Sample

Participants were pediatricians working in the conveniently selected twelve hospitals (six grade III and six grade II) in four major cities of Guangxi province (a Southern Chinese province), the People’s Republic of China. Hospital systems in China follow a grading system. The higher the grade, the larger the hospital and the more sophisticated the facility is. Grade III hospitals are general or comprehensive hospital at national, provincial or city level (>500 beds); Grade II hospitals are hospitals of medium size at city, county and district level (between 100 and 500 beds); and level one hospitals are the township hospitals (<100 beds). The size and characteristics of the pediatric departments and the patient population within different levels of hospital differs according to the grade of the hospital. Although, selected conveniently, these hospitals should be representative of similar grade hospitals in South China.

### Data collection

A standardized Mandarin Chinese language questionnaire was used for data collection. Questionnaires were distributed through the director of pediatrics department in each of the participating hospitals. The director’s designate distributed a copy of the questionnaire to each pediatrician working in his or her department and requested them to put the completed questionnaire in a sealed envelope and drop the questionnaire in the designated box kept in the doctor’s office. Pediatricians needed to write down their name and contact telephone number in the questionnaire for further clarification and payment of incentives. Our study coordinator then collected the sealed questionnaire from each of the directors. For clarity on any unfinished questions, our study coordinator contacted the individual pediatrician by telephone. To compensate for their time, each participant was given a cash amount of RMB 100 ($15). The study was approved by the institutional review board of Guangxi Medical University (No. IRB-Int-2013 (315-1)).

### Questionnaire

The questionnaire was developed with reference to the questionnaires previously used by the investigators team in the United States [9] and in China [25]. The details of the questionnaire have been described elsewhere [26]. Briefly, the questionnaire obtained information on the subject’s demographic and medical training background, smoking behavior (smoker, non-smoker), counseling practices for smoking cessation and secondhand smoke (SHS) exposure to children and other relevant factors.

Pediatricians’ use of the 5 A’s for smoking cessation was assessed by asking pediatricians to estimate how often in the past 30 work days they had asked about the smoking status of their patients’ parents, advised smoking parents to quit, assessed smoking parents willingness to quit, assisted smoking parents with a quit plan, or arranged follow-up contact. Responses to the questionnaire items were recorded in a 5-point Likert scale with response categories of “Always, Often, Sometimes, Seldom or Never”. We defined responses of “always” or “often” as “routine practice” and “sometimes, seldom or never” as non-routine practice.

### Analyses

Two members of the research team coded each questionnaire and entered all data with Epidata 3.1, and then made a data consistency check. The SPSS 16.0 statistical package was used for data analysis. Multivariate logistic regression was used to calculate odds ratios with 95 % confidence interval (CI) for describing differences between pediatricians who routinely provided any of the 5A’s smoking cessation counseling (e.g., any of the A’s were part of their routine practice) and those who did not routinely provide any of the 5A’s smoking cessation counseling. A *p*-value of <0.05 (two-tailed) was considered statistically significant.

## Results

### Demographic and other characteristics

A total of 504/550 (92 %) of pediatricians surveyed completed questionnaires. Response rates were almost identical in all the hospitals. As shown in Table 1, the majority of respondents were female (64 %), non-smokers (83 %), and received 5 years of education at medical school (77 %). Fewer than half of the respondents had appropriate knowledge about health risk of smoking (47 %) and secondhand smoke (44 %). More than two thirds of the respondents (68 %) had not heard about third hand smoke. Eighty one percent of the subjects received no formal training on smoking cessation and 85 % had not heard about or read about the China smoking cessation guidelines. Only 26 % of pediatricians followed any of the 5A’s for smoking cessation counseling in their practice.

**Table 1:** Demographic and other characteristics of survey sample, Guangxi, China 2013 (*n* = 504)

Variables	*N*	%
Demographic and work environment characteristics		
*Gender*		
Male	182	36
Female	322	64
*Age*		
20-30	215	43
31-40	159	31
41-50	89	18
Above 50	41	8
*Physician type* ^*a*^		
Resident Physician	223	45
Attending Physician	151	30
Chief or Associate Chief Physician	130	25
*Number of years studied at medical school* ^b^		
5 Years	388	77
More than 5 years	116	23
Tobacco use related characteristics		
*Smoking status*		
Current smokers	82	17
Non-smokers	400	83
*Heard about e-cigarettes*		
No	178	35
Yes	326	65
*Heard about third hand smoke*		
No	342	68
Yes	162	32
*Received cigarettes as gift or gave cigarettes as gifts to others*		
No	423	84
Yes	81	16
Hospital policy characteristics		
*Have smoke-free policy in the hospital*		
No policy	8	2
Have policy	496	98
*Hospital have any policy to advise smokers to quit*		
No	219	43
Yes	285	57
Training and work attitudes		
*Received formal training in smoking cessation*		
No	399	81
Yes	96	19
*Have read China smoking cessation guidelines*		
No/never heard	427	85
Yes	77	15
*Have read international (i.e. US, UK) smoking cessation guidelines*		
No/never heard	468	93
Yes	36	7
*Believe that it is pediatricians professional responsibility to discuss smoking cessation*		
No	275	55
Yes	229	45
*Level of confidence discussing smoking cessation or SHS exposure reduction with patients’ parents*		
Not at all confident	66	13
Somewhat confident	316	63
Very confident	122	24
*Beliefs regarding effectiveness of physician counseling for smoking cessation*		
Disagree/strongly disagree	200	40
Agree/strongly agree	304	60
*Beliefs regarding effectiveness of pharmacological treatment for smoking cessation*		
Disagree/strongly disagree	238	47
Agree/strongly agree	266	53
*Appropriate knowledge about health risk of smoking*		
No	267	53
Yes	237	47
*Appropriate knowledge about health risk of secondhand smoking*		
No	284	56
Yes	220	44
*Followed any of the 5As*		
Always or often	131	26
Otherwise	373	74

Notes: Due to the missing values in some variables, the total number may not equal to the same

^a^Resident physicians are medical graduates who works in the department of pediatrics under the supervision of fully licensed physicians (i.e. Attending or Chief Physicians). Attending physicians has completed residency and practices medicine in the hospital, who can also supervise resident physician. Chief or Associate Chief Physicians are the most senior physician with management responsibility

^b^In China, the length of medical education is for 5 years or 6-8 years, depending on the University one attends

### Patterns of 5A’s cessation counselling practices among Chinese pediatricians

Table 2 shows the patterns of 5A’s cessation counseling practices among Chinese pediatricians. When pediatricians were asked about their use of the 5A’s, 12.9 % reported they “always or often” asked about household members who smoke, and 22.4 % reported they “always or often” advised smoking parents to quit. Pediatricians were even less likely to indicate use of other effective practices, such as assessing smoking parents’ willingness to quit (5.8 %), assisting with a quit plan (5.8 %), or making follow-up arrangements (3.8 %).

**Table 2:** Patterns of 5A’s cessation counseling practices (always or often) among pediatricians, Guangxi, China 2013

Variables	Asked about household members who smoke *n* (%)	Advised to quit *n* (%)	Assessed willingness to quit *n* (%)	Assisted with a quit plan *n* (%)	Arranged follow-up contact *n* (%)
Total	65 (12.9)	113 (22.4)	29 (5.8)	29 (5.8)	19 (3.8)
Gender					
Male	17 (9.3)	36 (19.8)	11 (6.0)	11 (6.0)	6 (3.3)
Female	48 (14.9)	77 (23.9)	18 (5.6)	18 (5.6)	13 (4.0)
Ages					
20-30	33 (15.3)	48 (22.3)	14 (6.5)	12 (5.6)	9 (4.2)
31-40	18 (11.3)	36 (22.6)	8 (5.0)	7 (4.4)	6 (3.8)
41-50	6 (6.7)	19 (21.3)	5(5.6)	3 (3.4)	2 (2.2)
Above 50	8 (19.5)	10 (24.4)	2 (4.9)	7 (17.1)	2 (4.9)
Physician type					
Resident Physician	34 (15.2)	49 (22.0)	15 (6.7)	11 (4.9)	9 (4.0)
Attending Physician	17 (11.3)	35 (23.2)	7 (4.6)	12 (7.9)	6 (4.0)
Chief or Associate Chief Physician	14 (10.8)	29 (22.3)	7 (5.4)	6 (4.6)	4 (3.1)
Number of years studied at medical school					
5 Years	44 (11.3)	85 (21.9)	22 (5.7)	24 (6.2)	16 (4.1)
More than 5 years	21 (18.1)	28 (24.1)	7 (6.0)	5 (4.3)	3 (2.6)
Smoking status					
Current smoker	2 (2.4)	10 (12.2)	2 (2.4)	4 (4.9)	2 (2.4)
Nonsmoker	58 (14.5)	98 (24.5)	26 (6.5)	25 (6.3)	17 (4.3)
Heard about e-cigarettes					
No	20 (11.2)	28 (15.7)	7 (3.9)	8 (4.5)	5 (2.8)
Yes	45 (13.8)	85 (26.1)	22 (6.7)	21 (6.4)	14 (4.3)
Heard about third hand smoke					
No	40 (11.7)	65 (19)	18 (5.3)	18 (5.3)	11 (3.2)
Yes	25(15.4)	48 (29.6)	11 (6.8)	11 (6.8)	8 (4.9)
Received cigarettes as gift or gave cigarettes as gifts to others					
No	56 (13.2)	95 (22.5)	23 (5.4)	25 (5.9)	14 (3.3)
Yes	9 (11.1)	18 (22.2)	6 (7.4)	4 (4.9)	5 (6.2)
Have smoke-free policy in the hospital					
No policy	1 (12.5)	1 (12.5)	0 (0)	1 (12.5)	0 (0)
Have policy	64 (12.9)	112 (22.6)	29 (5.8)	28 (5.6)	19 (3.8)
Hospital have any policy to advise smokers to quit					
No	27 (12.3)	33 (15.1)	8 (3.7)	6 (2.7)	5 (2.3)
Yes	38 (13.3)	80 (28.1)	21 (7.4)	23 (8.1)	14 (4.9)
Received formal training in smoking cessation					
No	42 (10.5)	69 (17.3)	11 (2.8)	16 (4.0)	8 (2.0)
Yes	21 (21.9)	41 (42.7)	18 (18.8)	13 (13.5)	11 (11.5)
Have read China smoking cessation guidelines					
No/never heard	45 (10.5)	73 (17.1)	15 (3.5)	16 (3.7)	11 (2.6)
Yes	20 (26.0)	40 (51.9)	14 (18.2)	13 (16.9)	8 (10.4)
Have read international (i.e. US, UK) smoking cessation guidelines					
No/never heard	55 (11.8)	94 (20.1)	19 (4.1)	22 (4.7)	11 (2.4)
Yes	10 (27.8)	19 (52.8)	10 (27.8)	7 (19.4)	8 (22.2)
Believe that it is pediatricians professional responsibility to discuss smoking cessation					
No	24 (8.7)	47 (17.1)	7 (2.5)	9 (3.3)	4 (1.5)
Yes	41 (17.9)	66 (28.8)	22 (9.6)	20 (8.7)	15 (6.6)
Level of confidence discussing smoking cessation or SHS exposure reduction with patients’ parents					
Not at all confident	1 (1.5)	5 (7.6)	3 (4.5)	3 (4.5)	0 (0)
Somewhat confident	32 (10.1)	53 (16.8)	9 (2.8)	12 (3.8)	8 (2.5)
Very confident	32 (26.2)	55 (45.1)	17 (13.9)	14 (11.5)	11 (9.0)
Beliefs regarding effectiveness of physician counseling for smoking cessation					
Disagree/strongly disagree	27 (13.5)	40 (20.0)	9 (4.5)	10 (5.0)	8 (4.0)
Agree/strongly agree	38 (12.5)	73 (24.0)	20 (6.6)	19 (6.3)	11 (3.6)
Beliefs regarding effectiveness of pharmacological treatment for smoking cessation					
Disagree/strongly disagree	31 (13.0)	49 (20.1)	13 (5.5)	12 (5.0)	9 (3.8)
Agree/strongly agree	34 (12.8)	64 (24.1)	16 (6.0)	17 (6.4)	10 (3.8)
Appropriate knowledge about health risk of smoking					
No	30 (11.2)	57 (21.3)	12 (4.5)	17 (6.4)	9 (3.4)
Yes	35 (14.8)	56 (23.6)	17 (7.2)	12 (5.1)	10 (4.2)
Appropriate knowledge about health risk of secondhand smoking					
No	30 (10.6)	57 (20.1)	11 (3.9)	12 (4.2)	12 (4.2)
Yes	35 (15.9)	56 (25.5)	18 (8.2)	17 (7.7)	7 (3.2)
Parents are resistant to discuss about smoking					
Is a barrier	60 (12.7)	103 (21.9)	26 (5.5)	28 (5.9)	19 (4.0)
Is not a barrier	5 (15.2)	10 (30.3)	3 (9.1)	1 (3.0)	0 (0)
It is hard to find a time to talk with parents					
Is a barrier	58 (12.2)	99 (20.8)	22 (4.6)	24 (5.0)	15 (3.2)
Is not a barrier	7 (25.0)	14 (50.0)	7 (25.0)	5 (17.9)	4 (14.3)
Pediatricians are not trained to discuss smoking cessation with adults					
Is a barrier	59 (12.4)	104 (21.9)	24 (5.1)	24 (5.1)	16 (3.4)
Is not a barrier	6 (20.0)	9 (30.0)	5 (16.7)	5 (16.7)	3 (10.0)
Lack of a standard of care requiring pediatricians to provide smoking cessation or SHS exposure reduction intervention					
Is a barrier	53 (12.2)	88 (20.3)	22 (5.1)	18 (4.1)	11 (2.5)
Is not a barrier	12 (20.0)	25 (41.7)	7 (11.7)	11 (11.7)	8 (13.3)
Lack of insurance coverage for smoking cessation medication					
Is a barrier	53 (13.0)	84 (20.6)	20 (4.9)	21 (5.2)	12 (2.9)
Is not a barrier	12 (12.4)	29 (29.9)	9 (9.3)	8 (8.2)	7 (7.2)
It is hard to make system changes at our hospital					
Is a barrier	56 (12.3)	97 (21.2)	21 (4.6)	21 (4.6)	14 (3.1)
Is not a barrier	9 (19.1)	16 (34.0)	8 (17.0)	8 (17.0)	5 (10.6)
Not convinced that advice and/or available therapies would work					
Is a barrier	57 (13.1)	93 (21.3)	19 (4.4)	20 (4.6)	14 (3.2)
Is not a barrier	8 (11.8)	20 (29.4)	10 (14.7)	9 (13.2)	5 (7.4)

Note: Due to the missing values in some variables, the total number may not equal to the same

### Factors associated with 5A’s smoking cessation counselling practices among Chinese pediatricians

As shown in Table 3, factors that were associated with delivering 5A’s smoking cessation counseling included: being a never smoker (OR = 2.40), receiving formal training on smoking cessation (OR = 2.54), having read the China smoking cessation guidelines (OR = 2.11), being very confident (OR = 7.64) or somewhat confident (OR = 3.32) in discussing smoking or SHS exposure reduction with parents. Additionally, the following two variables significantly decreased the odds of often or always following the 5A’s guidelines: reporting: it is hard to find time to talk with parents.(OR = 0.32), and reporting lack of a standard of care requiring pediatricians to provide smoking cessation or SHS exposure reduction intervention (OR = 0.45).

**Table 3:** Prevalence and odds of providing any of the 5As by the Chinese pediatricians, Guangxi 2013

Variables	Followed any of the 5As (always or often) *n* (%)	Followed any of the 5As (Otherwise) *n* (%)	OR (95 % CI)
Gender			
Male (referent)	40 (22)	142 (78)	1
Female	91 (28)	231 (72)	1.31 (0.74,2.35)
Ages			
20-30 (referent)	61 (28)	154 (72)	1
31-40	38 (24)	121 (76)	0.57 (0.27,1.20)
41-50	19 (21)	70 (79)	0.44 (0.16,1.22)
Above 50	13 (32)	28 (68)	0.61 (0.19,1.93)
Physician type			
Resident Physician (referent)	62 (28)	161 (72)	1
Attending Physician	38 (25)	113(75)	0.96 (0.46,2.03)
Chief or Associate Chief Physician	31 (24)	99 (76)	1.48 (0.57,3.82)
Number of years studied at medical school			
5 Years (referent)	98 (25)	290 (75)	1
More than 5 years	33 (28)	83 (72)	1.14 (0.64,2.04)
Smoking status*			
Current smoker (referent)	11 (13)	71 (87)	1
Nonsmoker	114 (29)	286 (71)	2.40 (1.05,5.50)
Heard about e-cigarettes			
No (referent)	34 (19)	144 (81)	1
Yes	97 (30)	229 (70)	1.54(0.90,2.62)
Heard about third hand smoke			
No (referent)	80 (23)	262 (77)	1
Yes	51 (31)	111 (69)	1.24 (0.74,2.10)
Received cigarettes as gift or gave cigarettes as gifts to others			
No (referent)	112 (26)	311 (74)	1
Yes	19 (23)	62 (77)	0.87 (0.42,1.82)
Have smoke-free policy in the hospital			
No policy (referent)	2 (25)	6 (75)	1
Have policy	129 (26)	367 (74)	0.33 (0.05,2.30)
Hospital have any policy to advise smokers to quit			
No (referent)	46 (21)	173 (79)	1
Yes	85 (30)	200 (70)	1.25 (0.75,2.08)
Received formal training in smoking cessation**			
No (referent)	83 (21)	316 (79)	1
Yes	45 (47)	51 (53)	2.54 (1.38,4.67)
Have read China smoking cessation guidelines*			
No/never heard (referent)	90 (21)	337 (79)	1
Yes	41 (53)	36 (47)	2.11 (1.05,4.21)
Have read international (i.e. US, UK) smoking cessation guidelines			
No/never heard (referent)	111 (24)	357 (76)	1
Yes	20 (56)	16 (44)	1.88 (0.74,4.77)
Believe that it is pediatricians professional responsibility to discuss smoking cessation			
No (referent)	55 (20)	220 (80)	1
Yes	76 (33)	153 (67)	1.35 (0.83,2.21)
Level of confidence discussing smoking cessation or SHS exposure reduction with patients’ parents**			
Not at all confident (referent)	5 (8)	61 (92)	1
Somewhat confident	71 (22)	245 (78)	3.32 (1.17,9.44)
Very confident	55 (45)	67 (55)	7.64 (2.53,23.09)
Beliefs regarding effectiveness of physician counseling for smoking cessation			
Disagree/strongly disagree (referent)	51 (26)	149 (74)	1
Agree/strongly agree	80 (26)	224 (74)	0.67 (0.40,1.13)
Beliefs regarding effectiveness of pharmacological treatment for smoking cessation			
Disagree/strongly disagree (referent)	61 (26)	177 (74)	1
Agree/strongly agree	70 (26)	196 (74)	0.71 (0.43,1.19)
Appropriate knowledge about health risk of smoking			
No (referent)	68 (25)	199 (75)	1
Yes	63 (27)	174 (73)	0.77 (0.45,1.31)
Appropriate knowledge about health risk of secondhand smoking			
No (referent)	67 (24)	217 (76)	1
Yes	64 (29)	156 (71)	1.23 (0.72,2.08)
Parents are resistant to discuss about smoking			
Is not a barrier (referent)	10 (30)	23 (70)	1
Is a barrier	121 (26)	350 (74)	0.76 (0.29,2.01)
It is hard to find a time to talk with parents*			
Is not a barrier (referent)	15 (54)	13 (46)	1
Is a barrier	116 (24)	360 (76)	0.32 (0.11,0.92)
Pediatricians are not trained to discuss smoking cessation with adults			
Is not a barrier (referent)	10 (33)	20 (67)	1
Is a barrier	121 (26)	353 (74)	2.43 (0.72,8.22)
Lack of a standard of care requiring pediatricians to provide smoking cessation or SHS exposure reduction intervention*			
Is not a barrier (referent)	20(33)	40 (67)	1
Is a barrier	101 (23)	333 (77)	0.45 (0.21,0.98)
Lack of insurance coverage for smoking cessation medication			
Is not a barrier (referent)	31 (32)	66 (68)	1
Is a barrier	100 (25)	307 (75)	1.42 (0.68,2.97)
It is hard to make system changes at our hospital			
Is not a barrier (referent)	16 (34)	31 (66)	1
Is a barrier	115 (25)	342 (75)	1.32 (0.47,3.75)
Not convinced that advice and/or available therapies would work			
Is not a barrier (referent)	21 (31)	47 (69)	1
Is a barrier	110	326 (75)	0.94 (0.46,1.93)

Note: *CI* Confidence interval; *OR* Odds ratio.*P < 0.05, **P < 0.01

### Patterns of tobacco use reduction or cessation services provided by pediatricians

Table 4 shows patterns of tobacco use reduction or cessation services provided by pediatricians. Only one fifth of pediatricians always or often talked to smoking parents about secondhand smoke and its effect on child health. Pediatricians were far less likely to “always or often” refer smoking parents to a Quitline or some other smoking cessation services (3 %), suggest that they use some form of pharmacological support (3.4 %), or prescribe medications (i.e. nicotine replacement therapy) to help them quit smoking (0.4 %).

**Table 4:** Patterns of tobacco use reduction or cessation services provided by pediatricians

Types of cessation services provided	Always or often *n* (%)	Otherwise *n* (%)
Talked to them about secondhand smoke and its effect on health	101 (20.0)	403 (80.0)
Suggested that they should use some form of pharmacological support	17 (3.4)	487 (96.6)
Prescribed medications (patch, gum, inhaler, zyban, varenicline)	2 (0.4)	502 (99.6)
Referred to a Quitline or other available smoking cessation service	15 (3.0)	489 (97.0)

## Discussion

In this study, we found that few Chinese pediatricians reported implementing commonly recommended 5A’s smoking cessation practices for smoking parents. Overall, routinely advising smoking parents to quit was the most common practice, with practices pertaining to asking about household smokers, assessing smoking parents’ willingness to quit, assisting with a quit plan, and arranging follow-up contact showing much lower rates of endorsement.

Providing other tobacco use reduction or cessation services, such as, talking to smoking parents about SHS and its effect on health, referring smoking parents to a Quitline or other smoking cessation services, was also not common.

The smoking rate of pediatricians in the current study (17 %) is lower than the other recent reports among physicians. In a study 2007 by Abdullah et al., [25], overall current smoking prevalence among Chinese physician (Guangxi province) was 26 % (men, 35 %; women, 3 %). In another China nationwide study by Jiang et al. [27], the overall prevalence was 23 % (41 % for men and 1 % for women). The low rate in the current study is due to the larger proportion of female doctors (64 %) in the sample population.

In this study, few reported feeling very confident in their ability to deliver smoking cessation counseling, and more than four fifths of pediatricians reported not being trained to discuss smoking cessation with the parents. Pediatricians’ degree of confidence in delivering smoking cessation counseling appeared to be a significant factor in their use of the 5A’s counseling steps with smoking parents. Research had shown that pediatricians who received formal training in smoking cessation counseling reported higher levels of self-efficacy for smoking cessation [19] and provided more smoking cessation counseling to their patients [20]. A survey in China reported higher confidence to provide smoking cessation service among physicians who received training on tobacco use prevention and cessation [26]. At the same time, only less than half of pediatricians had appropriate knowledge about health risk of smoking and SHS, and about half of the pediatricians did not believe the fact that smoking cessation counseling and medication are effective in promoting smoking cessation. These knowledge gaps about the harms of smoking and SHS, and misconceptions about the effectiveness of counseling and medication may reflect the need for more trainings on SHS exposure reduction and smoking cessation counseling among Chinese pediatricians.

Our study found that the majority of pediatricians did not read the China smoking cessation guidelines, and fewer than half of respondents believed that it is the pediatrician’s professional responsibility to discuss smoking cessation with their patients’ parents. If pediatricians do not perceive smoking cessation counseling as highly relevant to their practice then their interest to read any guidelines is likely to be low. On the other hand, unlike the American Academy of Pediatrics [14], there were no recommendations made by the Chinese Pediatric Society (CPS) to follow clinical guidelines for smoking cessation. Also the guidelines were not widely disseminated to all physicians. We also found that lack of a standard of care requiring pediatricians to provide smoking cessation or SHS exposure reduction intervention was a factor to limited pediatricians’ use of the 5A’swith smoking parents. Better coordination among different Chinese medical professional societies is needed to promote the Chinese clinical practice guidelines for smoking cessation. Training pediatricians systematically on the CEASE program had led to an increase in the provision of cessation assistance in the United States [28, 29]. Similar approaches to train Chinese pediatricians should be considered to reduce children’s exposure to SHS from parental smoking.

The scarcity of Quitline, insurance coverage for NRT, or other smoking cessation programs available in China may have been part of the cause for Chinese Pediatrician’s lack of referral to other smoking cessation services or not prescribing any medications. Certain types of scattered smoking cessation services, including Quitline [30] and smoking cessation clinic [31], are now available only in the major cities of Beijing and Shanghai with very limited cessation programs available in other cities. Although the reach and effectiveness of these programs are not known, low cost Quitline [32] and clinic based smoking cessation services [33] were effective among the Hong Kong Chinese. To facilitate referral by clinicians and support those smokers who want to quit smoking, it may be important to promote organized smoking cessation services within the healthcare system and through other population-based programs.

### Limitations

Several factors may limit the generalizability of the findings. First, the sample may not be representative of the whole pediatrician population in China. Although it is expected that the characteristics of pediatricians working in all the similar grade (grade III or grade II) level hospitals would be similar, there may be regional variations. However, responses to key items did not differ as a function of hospital type or physician type so this is unlikely to have affected the results. Second, all the responses are self-reported behavior or estimates made by the pediatricians without any validation from the clinical chart or patients’ parents. It is possible that pediatricians overestimated the frequency of preventive services that they had actually provided. Third, we assessed 5A’s utilization based on the presence of any of the 5A’s in their routine practice. This was the minimum expectation of 5A’s utilization, which have overestimated the 5A’s practice in the Chinese pediatric setting. Fourth, although we have assessed the implementation of 5A’s by pediatricians, in the current Chinese healthcare delivery system which lacks organized smoking cessation programs and insurance coverage for cessation medications, there are difficulties in fully implementing the 5A’s by healthcare providers. At the same time, the current medical curriculum or the specialist training program do not have a training component for smoking cessation, which leave pediatricians to seek for training opportunities by their own.

## Conclusion

The findings from this study suggest that many pediatricians are not addressing the SHS exposure of their pediatric patients – missing an important opportunity for intervention. Pediatricians appear to lack skills and training to implement smoking cessation counseling with smoking parents. Training pediatricians in smoking cessation counseling will build their capacity and, hence, the level of confidence to provide smoking cessation and SHS exposure reduction counseling to parents. Adopting evidence-based system strategies for increasing implementation of all 5A’s, such as electronic reminder systems to provide the 5 A’s, and availability of resources and feedback to pediatricians [18, 34] could facilitate pediatrician delivery of the 5A’s in routine practice. A multi-faceted approach would include creation of a smokefree hospital environment and culture. This approach should include, at least, the following components: requiring smoking status as a vital sign to be noted on the patient registration form; the provision of designated smoking cessation team composed of nurses and/or patient educators; and availability and insurance coverage for nicotine replacement therapy or other pharmacological products [35–37].

## Abbreviations

CEASE: Clinical Effort Against Secondhand Smoke Exposure
OR: Odds Ratio
SHS: Second-hand Smoke

## References

[1] Wang CP, Xu XF, Ma SJ, Mei CZ, Wang JF, Chen AP, Yang GH (2008). The current status of passive smoking in Chinese families and associated factors. Zhonghua Yu Fang Yi Xue Za Zhi.

[2] Yolton K, Dietrich K, Auinger P, Lanphear BP, Hornung R (2005). Exposure to environmental tobacco smoke and cognitive abilities among U.S.children and adolescents. Environ Health Perspect.

[3] DiFranza JR, Lew RA (1996). Morbidity and mortality in children associated with the use of tobacco products by other people. Pediatrics.

[4] American Academy of Family Physicians (1992). AAFP age charts for periodic health examinations: 13 to 18 years. Am Fam Physician.

[5] Winickoff JP, Hillis VJ, Palfrey JS, Perrin JM, Rigotti NA (2003). A smoking cessation intervention for parents of children who are hospitalized for respiratory illness: the stop tobacco outreach program. Pediatrics.

[6] Yang GH (2011). Global Adult Tobacco Survey (GATS) China 2010 Country Report.

[7] Klein JD, Portilla M, Goldstein A, Leininger L (1995). Training pediatric residents to prevent tobacco use. Pediatrics.

[8] Newacheck PW, Stoddard JJ, Hughes DC, Pearl M (1998). Health insurance and access to primary care for children. N Engl J Med.

[9] Winickoff JP, Buckley VJ, Palfrey JS, Perrin JM, Rigotti NA (2003). Intervention with parental smokers in an outpatient pediatric clinic using counseling and nicotine replacement. Pediatrics.

[10] Frankowski BL, Secker-Walker RH, National Cancer Institute (1994). Pediatricians’ role in smoking prevention and cessation. Tobacco and the Clinician: Interventions for Medical and Dental Practice.

[11] Cluss PA, Moss D (2002). Parent attitudes about pediatricians addressing parental smoking. Ambul Pediatr.

[12] Abdullah AS, Hua F, Xia X, Hurlburt S, Ng P, MacLeod W, Siegel M, Griffiths S, Zhang Z. Second hand smoke exposure and household smoking bans in Chinese families: a qualitative study. Health Soc Care Community. 2012; 20(4):356-64. 10.1111/j.1365-2524.2011.01035.x 22029412

[13] Best D, Moss DA, Winickoff JP, Simpson L (2006). Ambulatory Pediatric Association policy on tobacco. Ambul Pediatr.

[14] Committee on Substance Abuse (2001). American Academy of Pediatrics: tobacco’s toll: implications for the pediatrician. Pediatrics.

[15] American Academy of Pediatrics (AAP) Committee on Environmental Hazards: Involuntary smoking – a hazard to children. Pediatrics 1986;77(5):755–757. Retrieved from [http://pediatrics.aappublications.org/content/77/5/755.short] 3703641

[16] Fiore MC, Bailey WC, Cohen SJ, Dorfman SF, Fox BJ, Goldstein MG (2000). A clinical practice guideline for treating tobacco use and dependence. JAMA.

[17] American Academy of Pediatrics Committee on Substance Abuse (2001). Tobacco’s toll: implications for the pediatrician. Pediatrics.

[18] Rosen LJ, Noach MB, Winickoff JP, Hovell MF (2012). Parental smoking cessation to protect young children: a systematic review and meta-analysis. Pediatrics.

[19] Quinn VP, Steven VJ, Hollis JF (2005). Tobacco-cessation services and patient satisfaction in nine nonprofit HMOs. Am J Prev Med.

[20] Katz DA, Muehlenbruch DR, Brown RL, Fiore MC, Baker TB (2004). AHRQ Smoking Cessation Guideline Study Group. Effectiveness of implementing the Agency for Healthcare Research and Quality smoking cessation clinical practice guideline: a randomized, controlled trial. J Natl Cancer Inst.

[21] Quinn VP, Hollis JF, Smith KS (2009). Effectiveness of the 5-As tobacco cessation treatments in nine HMOs. J Gen Intern Med.

[22] Thorndike AN, Rigotti NA, Stafford RS, Singer DE (1998). National patterns in the treatment of smokers by physicians. JAMA.

[23] Simmons VN, Litvin EB, Unrod M, Brandon TH (2011). Oncology healthcare providers’ implementation of the 5 A’s model of brief intervention for smoking cessation: patients’ perceptions. Patient Educ Couns.

[24] WHO Collaborating Centre for tobacco or health, Chinese Center for Disease Control and Prevention, Chinese Association on Tobacco Control, Specialized Subcommittee on Tobacco Control in hospital (2008). Chinese clinical practice guidelines for smoking cessation in 2007. International Journal of Respiration.

[25] Zhou J, Abdullah AS, Pun VC, Huang D, Lu S, Luo S (2010). Smoking status and cessation counseling practices among physicians, Guangxi, China, 2007. Prev Chronic Dis.

[26] Huang K, Abdullah AS, Liao J, Huo H, Yang L, Zhang Z, Winickoff JP, Nong G (2015). Chinese pediatrician beliefs about counseling and medications for parents who smoke: a survey in southern China. Tob Induc Dis.

[27] Jiang Y, Ong MK, Tong EK, Yang Y, Nan Y, Gan Q (2007). Chinese physicians and their smoking knowledge, attitudes, and practices. Am J Prev Med.

[28] Winickoff JP, Park ER, Hipple BJ, Berkowitz A, Vieira C, Friebely J, Rigotti NA (2008). Clinical effort against secondhand smoke exposure: development of framework and intervention. Pediatrics.

[29] Winickoff JP. Pediatrician-led program increases provision of smoking cessation support, boosts quit rates among parents. Innov Med 2011. Accessed on 15 May 2012 at [http://innovations.ahrq.gov/content.aspx? id = 2580]

[30] Chen WL, Xiao D, Henderson S, Zhao L, Jing H, Wang C (2013). Characteristics of callers accessing the tobacco cessation quitline in mainland China. Biomed Environ Sci.

[31] Wu L, He Y, Jiang B, Zuo F, Liu Q, Zhang L, Zhou C, Liu M, Chen H (2014). Predictors for ‘successful quitting smoking’ among males carried out in a smoking cessation clinic. Zhonghua Liu Xing Bing Xue Za Zhi.

[32] Abdullah AS, Lam TH, Chan SC, Hedley AJ (2004). Which smokers use the smoking cessation Quitline in Hong Kong and how effective is the Quitline?. Tob Control.

[33] Abdullah AS, Hedley AJ, Chan SC, Ho WWN, Lam TH (2004). Establishment and evaluation of a smoking cessation clinic in Hong Kong: a model for the future service provider. J Public Health Med.

[34] Andrews JO, Tingen MS, Waller JL, Harper RJ (2011). Provider feedback improves adherence with AHCPR smoking cessation guideline. Prev Med.

[35] Fiore M, Jorenby D, Schensky A, Smith S, Bauer R, Baker T (1995). Smoking status as the new vital sign: effect on assessment and intervention in patients who smoke. Mayo Clin Proc.

[36] Hollis JF, Bills R, Whitlock E, Stevens VJ, Mullooly J, Lichtenstein E (2002). Implementing tobacco interventions in the real world of managed care. Tob Control.

[37] Solberg LI, Davidson G, Alesci NL, Boyle RG, Magnan S (2002). Physician smoking- cessation actions: are they dependent on insurance coverage or patients?. Am J Prev Med.

